# Acute Effects of Percussive Massage Intensity on Change-of-Direction Performance, Vertical Jump Kinetics, and Neuromuscular Performance Across Morning and Evening Sessions in Trained Male Football Players

**DOI:** 10.3390/medicina62030439

**Published:** 2026-02-26

**Authors:** Özgür Eken, İlinsu Demiralp, Birgül Arslanoğlu, Tahir Volkan Aslan, İsmihan Eken, Burak Yagin, Monira I. Aldhahi

**Affiliations:** 1 Department of Physical Education and Sport Teaching, Faculty of Sports Sciences, Inonu University, Malatya 44280, Turkey; ozgur.eken@inonu.edu.tr (Ö.E.); ismihanyigit.1@gmail.com (İ.E.); 2 Department of Sports Management, Faculty of Sports Sciences, Istanbul Aydin University, Istanbul 34295, Turkey; ilinsudemiralp@aydin.edu.tr; 3 Department of Humanities and Social Sciences, Faculty of Sciences and Letters, Istanbul Technical University, Istanbul 34469, Turkey; demirkolb@itu.edu.tr; 4 Department of Travel, Tourism and Entertainment Services, Erdemli Vocational School, Mersin University, Erdemli, Mersin 33740, Turkey; tahirvolkanaslan@gmail.com; 5 Department of Biostatistics and Medical Informatics, Faculty of Medicine, Inonu University, Malatya 44280, Turkey; 6 Department of Rehabilitation Sciences, College of Health and Rehabilitation Sciences, Princess Nourah bint Abdulrahman University, P.O. Box 84428, Riyadh 11671, Saudi Arabia

**Keywords:** percussive massage, massage gun, vibration therapy, circadian rhythm, explosive power, warm-up, football, dose–response

## Abstract

*Background and Objectives:* Percussive massage devices (PMDs) are increasingly used as warm-up tools to enhance neuromuscular performance; however, evidence regarding the optimal intensity and its interaction with circadian variation remains limited. This study examined the acute effects of two percussive massage intensities (low: 28 Hz; moderate: 35 Hz) compared with no massage on change-of-direction (COD) performance, vertical jump kinetics, and neuromuscular variables in trained male football players across morning and evening sessions. *Materials and Methods:* Eighteen trained male football players completed a randomized, counterbalanced crossover design involving three protocols (no massage, 28 Hz, and 35 Hz) performed in both morning (09:00–11:00) and evening (17:00–19:00) sessions following a standardized warm-up protocol. COD performance (T-Test and Illinois COD Test), countermovement jump height, and model-derived kinetic variables were assessed. *Results:* Significant main effects of the protocol were observed for T-test performance, jump height, velocity-related variables, and kinetic outcomes (*p* < 0.001; large effect sizes), with both percussive massage intensities outperforming the no-massage condition. Significant protocol × time-of-day interactions emerged for jump height, force, and impulse-related variables (*p* < 0.05), indicating greater morning-specific benefits following moderate-intensity (35 Hz) massage. The Illinois COD Test showed no significant protocol-related changes. *Conclusions:* Acute percussive massage enhances COD performance and vertical jump-related outcomes in trained football players. While both intensities are effective for general performance enhancement, moderate-intensity massage (35 Hz) appears to be more effective for optimizing force–time characteristics and attenuating morning-related performance decrements. These findings support the inclusion of intensity- and time-specific percussive massage strategies in warm-up routines.

## 1. Introduction

Warm-up protocols aimed at optimizing athletic performance constitute a fundamental component of physiological preparation prior to training sessions and competitive events [1,2]. While traditional manual massage has been associated with acute performance decrements [3], percussive massage (PM) has emerged as a distinct modality within pre-performance routines. Recent evidence suggests that PM may support acute preparation by enhancing range of motion without impairing neuromuscular function, potentially due to the superimposed vibration stimulus [4,5]. Massage, defined as the application of controlled mechanical manipulations to soft tissues [6], is commonly used to support muscular recovery, reduce injury risk, and facilitate acute tissue readiness [7]. Depending on application context and timing, massage may exert both relaxing and stimulating effects that influence athletic performance before, during, or after exercise [8].

In recent years, handheld percussive massage devices (PMDs) have gained substantial popularity in therapeutic and athletic settings [9]. These devices are used across multiple contexts, including preactivity preparation, postexercise recovery, and management of myofascial dysfunction. PMDs are commercially available in various models for self-application or clinician-supervised use (e.g., Hypervolt^®^ (Hyperice Inc., Irvine, CA, USA), TheraGun^®^ (TheraBody, LLC, Los Angeles, CA, USA) [10], operating across a wide range of frequencies and utilizing interchangeable applicator heads selected according to the treated anatomical region [10]. Experimental evidence suggests that PMDs may enhance viscoelastic tissue properties by reducing passive muscle stiffness and viscosity, particularly when vibration is applied within the 5–300 Hz range [5,7,11]. Despite their widespread adoption, PMD applications in the literature remain largely limited to short-term and single-session protocols, and no standardized guidelines currently exist regarding optimal application duration or frequency intensity.

Current evidence indicates that traditional massage can reduce delayed-onset muscle soreness and enhance joint range of motion in the short term; however, it does not appear to yield significant improvements in performance indicators such as strength, jump, sprinting, endurance, and fatigue. Similarly, both whole-body and localized vibration applied via vibrating foam rollers have been shown to support range of motion; however, evidence regarding their effects on performance remains unconvincing [12,13,14]. Consistent with the inconsistencies observed in traditional massage literature, several studies examining PMDs have reported no significant changes in neuromuscular recruitment or maximal force production capacity [7,15]. Nevertheless, due to their ability to enhance flexibility without compromising neuromuscular output, PMDs are suggested as a viable adjunct within pre-performance warm-up protocols to optimize tissue compliance [5,7]. This mechanical superimposition of vibration may uniquely influence the tonic vibration reflex, potentially distinguishing its physiological impact from manual manipulation [16]. PMD applications are largely confined to short-term and single-session protocols, and currently, no standardized approach exists regarding the optimal application duration, frequency, and intensity. The selection of 28 Hz and 35 Hz was based on evidence indicating that vibration frequencies around 30–40 Hz optimally stimulate muscle spindle activity and the tonic vibration reflex, supporting neuromuscular activation and force production in acute settings [4,16].

While extensive research has examined PMD in the context of recovery and fatigue management [17], its efficacy as an acute priming modality for enhancing kinetic output and neuromuscular performance remains less defined. Conflicting results in the literature regarding acute performance changes may be attributed to variations in vibration frequency and application time, highlighting the need for dose–response investigations [5,15,18].

Conversely, a systematic review addressing percussive massage interventions suggested that when applied as an acute pre-exercise modality, these tools may enhance neuromuscular activation and mechanical output [4]. Specifically, these findings indicate that short-duration applications (e.g., 2–5 min per muscle group) can improve flexibility and force production without the performance deficits typically associated with manual massage. However, the review also highlighted that most existing studies focus on isolated muscle groups rather than complex athletic movements like change-of-direction (COD) or vertical jump kinetics. This lack of evidence regarding how specific PMD intensities (Hz) interact with performance timing (e.g., morning vs. evening) forms the core rationale of our study [4,5,15].

Furthermore, athletic performance is profoundly influenced by circadian rhythms, which regulate essential physiological functions through endogenous molecular clocks [19]. These biological rhythms drive diurnal variations in core body temperature, hormonal homeostasis, and neuromuscular efficiency, typically resulting in a performance peak during the late afternoon [20]. Specifically, muscle force production and anaerobic capacity often exhibit a ‘morning deficit’ due to lower body temperatures and reduced neural drive early in the day [20,21]. However, it remains unclear whether acute priming interventions, such as percussive massage devices (PMDs), can effectively mitigate these diurnal fluctuations or further enhance evening peak performance. Understanding how PMD intensity interacts with these biological rhythms is essential for developing time-of-day-specific warm-up strategies to optimize neuromuscular readiness.

Extensive literature has established that circadian rhythms profoundly influence athletic performance through daily oscillations in physiological and behavioral function. For instance, Drust et al. (2005) conducted a comprehensive review demonstrating that circadian rhythms modulate sensory–motor, perceptual, cognitive, and neuromuscular variables, with peak performance typically occurring in the late afternoon or evening [22]. Similarly, Chtourou and Souissi (2012) synthesized evidence showing circadian effects on anaerobic power, endurance, and muscle strength, attributing evening superiority to elevated core body temperature and faster nerve conduction velocity [23]. More recent studies, such as Pallarés et al. (2014), examined neuromuscular and sprint performance in swimmers, linking morning decrements to lower body temperature and altered hormonal profiles (e.g., higher cortisol inhibiting force production) [24]. Racinais (2010) further explored environmental influences, finding that heat exposure exacerbates morning–afternoon differences [25], while a 2023 narrative review by Nobari et al. (2023) emphasized circadian rhythm’s role in hormonal regulation, immune function, and injury prevention [20]. This review highlights how circadian synchronization with photonic (light) and non-photonic stimuli (e.g., exercise and feeding) optimizes performance, with disruptions leading to impaired outcomes. Additionally, Castelli et al. (2024) underscored the bidirectional relationship between circadian rhythms and physical activity, noting that chronotype (morningness/eveningness) and sleep hygiene influence athletic performance [26].

Nevertheless, despite these potential benefits, inconsistencies persist regarding the acute effects of PMDs on performance. The literature highlights a notable lack of standardized protocols, particularly concerning the interaction between application duration and frequency intensity, which has led to divergent outcomes and limited the generalizability of results [27,28]. Therefore, more comprehensive research based on controlled, systematic, and comparative experimental designs is required to elucidate the influence of different percussive intensities on acute neuromuscular and kinetic outcomes.

Taken together, the literature highlights three key limitations: (i) a lack of standardized PMD protocols, particularly regarding frequency intensity and application duration; (ii) a predominance of recovery-oriented rather than performance-oriented outcomes; and (iii) a scarcity of studies examining PMD effects within a circadian framework. Therefore, the present study aimed to systematically investigate the acute effects of different percussive massage protocols (no massage, low-intensity [28 Hz], and moderate-intensity [35 Hz]) on COD performance, vertical jump performance, and kinetic variables in trained male football players, assessed during morning and evening sessions. It was hypothesized that (1) percussive massage would enhance neuromuscular and kinetic performance compared with a no-massage condition, and (2) moderate-intensity PM would elicit greater performance benefits, particularly during morning sessions characterized by circadian-related performance decrements.

## 2. Materials and Methods

### 2.1. Participants

A priori sample size estimation was conducted using G*Power 3.1 software to detect interaction effects in a repeated-measures ANOVA design. Based on an assumed effect size (partial η^2^ = 0.20), an alpha level of 0.05, statistical power of 0.80, three conditions, two measurement time points, and a sphericity correction of ε = 1, the analysis indicated that a minimum of 15 participants was required (actual power = 86.96%).

Eighteen male football players from the youth development squad of Malatyaspor, a club competing in the Turkish Süper Lig youth development system, participated in the study and completed all experimental conditions. Prior to inclusion, all athletes underwent medical screening based on self-reported health history to exclude individuals with acute or chronic musculoskeletal injuries, neurological disorders, or medical conditions that could affect their neuromuscular performance.

The inclusion criteria were as follows: (i) male athletes aged 18–25 years, (ii) possession of an active football license with at least two years of systematic football training experience, (iii) regular participation in football training at least three times per week, and (iv) willingness to attend both morning and evening testing sessions. Participants who were unable to complete all experimental sessions or who reported illness, pain, or discomfort during the testing period were excluded from the final analysis.

The demographic and physical characteristics of the participants are presented as mean ± standard deviation values. The participants had a mean age of 21.22 ± 1.93 years, height of 178.72 ± 7.51 cm, body mass of 69.06 ± 9.30 kg, body mass index of 21.56 ± 2.03 kg/m^2^, and training experience of 3.61 ± 1.20 y. Chronotype assessment indicated an intermediate profile (Morningness–Eveningness Questionnaire score: 49.17 ± 6.54).

All participants were fully informed about the study procedures, potential risks, and benefits prior to the data collection. The study protocol was approved by the Ethics Committee of Inonu University (14 October 2025, Approval No: 2025/8398), and written informed consent was obtained from all participants in accordance with the principles of the Declaration of Helsinki.

### 2.2. Experimental Design

This study utilized a randomized, counterbalanced, crossover design with repeated measures to examine the acute effects of different percussive massage intensities on neuromuscular performance at different times of the day.

The independent variables were the experimental protocol (three conditions: no massage [NM; passive control], low-intensity percussive massage [L1; 28 Hz], and moderate-intensity percussive massage [L3; 35 Hz]) and time of day (morning: 09:00–11:00; evening: 17:00–19:00). The dependent variables included change-of-direction (COD) performance (T-Test and Illinois COD Test), countermovement jump height, and kinetic variables derived from force–velocity analysis using the My Jump Lab application (impulse, peak force, relative force, take-off velocity, mean velocity, derived kinetic output [power], and flight time).

Each of the 18 participants completed all six experimental conditions (three protocols × two time points), serving as their own controls. The order of the experimental conditions was randomized using a computer-generated random sequence and counterbalanced across participants to minimize order effects. All testing sessions were conducted on non-training days or at least 24 h after the last training session. A minimum washout period of 48 h was enforced between sessions to eliminate the potential carryover effects. This interval was considered sufficient, as the physiological and performance-related effects of percussive massage are predominantly acute, with neuromuscular responses typically returning to baseline within 30–60 min post-application [29,30].

**Figure 1:**
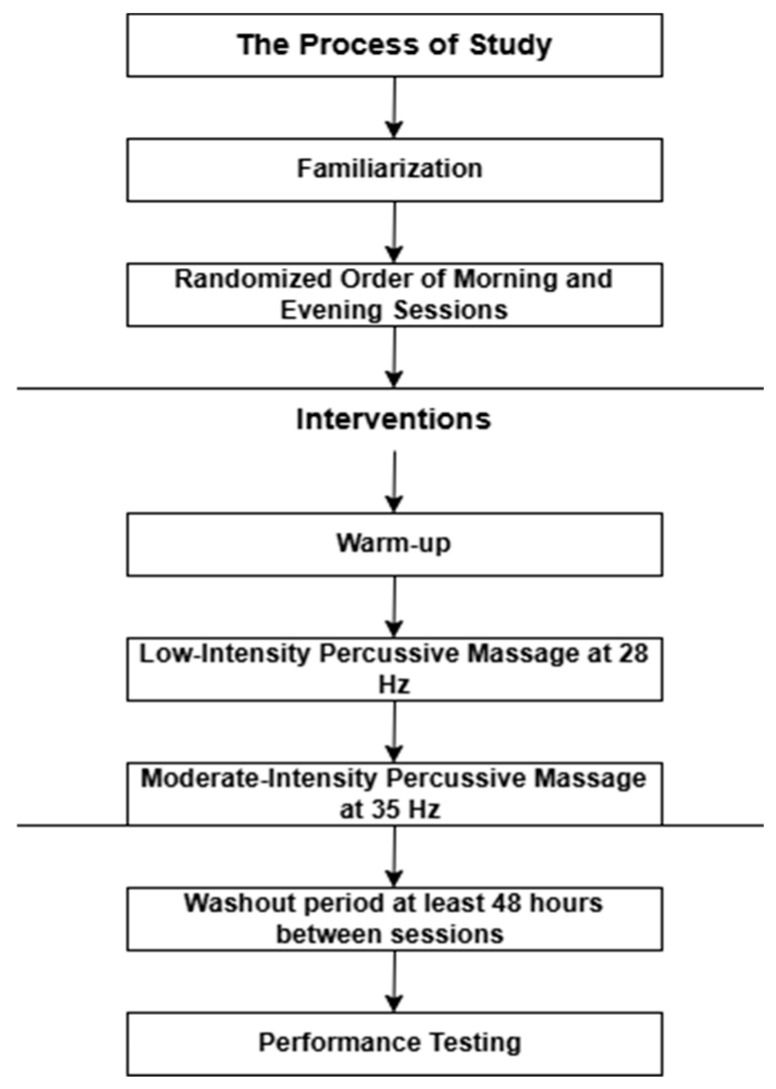
Schematic representation of the study procedures.

Due to the tactile nature of percussive massage, full blinding to the massage intensity conditions was not feasible. However, the participants were blinded to the study hypotheses and expected outcomes of each intensity level. The investigator applying the intervention was aware of the assigned protocols but was blinded to the specific study hypotheses. The no-massage condition consisted of passive seated rest of equivalent duration (5 min), allowing the isolation of baseline circadian performance variations. The overall study procedure is shown in Figure 1.

### 2.3. Intervention Protocols

All testing sessions were conducted in a controlled laboratory environment maintained at a constant ambient temperature (22–26 °C). Participants were instructed to maintain their habitual daily activities throughout the study period but refrain from strenuous physical exercise for at least 24 h prior to each testing session. In addition, they were asked to avoid food and carbonated beverages for a minimum of 2 h before testing. All experimental conditions were completed in a randomized order, maintaining a 48 h washout period between the morning (09:00–11:00) and evening (17:00–19:00) testing sessions. This interval ensured that the participants had fully recovered from the physical demands of the previous assessment and the acute stimuli of the percussive intervention. All testing sessions were conducted on non-training days or at least 24 h after the participants’ last training session to minimize the influence of fatigue.

The standardized warm-up protocol lasted approximately 10 min and was structured according to the RAMP (Raise, Activate, Mobilize, Potentiate) framework to ensure optimal physiological preparation [31,32]. The protocol consisted of three distinct phases: (1) Raise phase (4 min): low-intensity jogging at a self-selected pace (RPE 3–4), maintaining a heart rate of 60–70% HRmax (monitored via Polar H10); (2) Activation and mobilization phase (4 min): target-specific dynamic movements including 10 repetitions of bodyweight squats, walking lunges, lateral lunges, and dynamic glute bridges to enhance lower-limb joint range of motion; and (3) Potentiation phase (2 min): progressive intensity movements including high knees, butt kicks, and three sub-maximal 20 m sprints (at 70%, 80%, and 90% of perceived maximum speed). This comprehensive approach was designed to elevate core temperature and neuromuscular readiness without inducing fatigue, ensuring that any subsequent performance changes observed with PMD interventions were additive to a professional-standard warm-up baseline [1].

Immediately after completing the standardized warm-up, the participants underwent the assigned intervention for that session. All percussive massage applications were performed in a dedicated laboratory under standardized hygienic conditions. The massage device and treatment surfaces were cleaned and disinfected before each use of the device. To ensure consistency and minimize inter-operator variability, all massage applications were performed by the same trained investigator throughout the study.

Percussive massage was administered bilaterally using a Hypervolt 2 Pro handheld massage gun (Hyperice Inc., Irvine, CA, USA). The total intervention duration was 5 min (2.5 min per leg). Targeted muscle groups included the quadriceps (vastus lateralis, vastus medialis, and rectus femoris), hamstrings (biceps femoris, semitendinosus, and semimembranosus), gluteal muscles (gluteus maximus), and calf muscles (gastrocnemius medialis and lateralis). A round (ball) attachment head was used throughout the study to provide broad tissue coverage and consistent contact. The application was standardized as follows: approximately 35–40 s was allocated to each major muscle group per leg, with brief transitions ensuring a total of 2.5 min per leg. The device was moved continuously in longitudinal strokes (proximal to distal and return) at a slow, controlled speed of approximately 3–5 cm/s, avoiding prolonged static pressure at any single point. Light-to-moderate pressure was maintained (subjective rating 3–5 on a 0–10 scale, where 0 = no pressure and 10 = maximum tolerable), applied by the same experienced investigator across all sessions and for all participants. To verify consistency of the applied pressure, the investigator was familiarized with the protocol prior to data collection and practiced maintaining the predefined pressure range during pilot applications.

The selection of percussive frequencies was based on the device’s operational range and established protocols in the recent literature. According to the manufacturer’s specifications, Hypervolt 2 Pro offers five intensity levels ranging from 28 to 45 Hz. In this study, Level 1 (28 Hz) was classified as low-intensity and Level 3 (35 Hz) as moderate-intensity, representing the lower and middle tiers of the device functional spectrum. These specific frequencies were chosen as they align with previous investigations suggesting that vibration frequencies between 30 and 40 Hz are optimal for stimulating neuromuscular responses and enhancing tissue compliance without inducing premature muscle fatigue [18,33].

Although the device allows operation up to 45 Hz, higher frequencies were not included to avoid potential overstimulation or acute neuromuscular fatigue responses reported at elevated vibration intensities. Therefore, 35 Hz was selected as an evidence-informed moderate intensity within the proposed 30–40 Hz neuromuscular activation window rather than merely representing a mid-tier device setting.

After completing the assigned protocol, the participants performed the performance testing battery in a fixed order. The first performance assessment (T-test) commenced approximately 60 s after completion of the intervention to standardize acute potentiation latency across participants.

Immediately after completing the assigned protocol, the participants performed the performance testing battery in a fixed order. A standardized passive recovery period of approximately 2 min was provided between the consecutive tests. All assessments were conducted by the same investigators, who were blinded to the massage intensity conditions to ensure consistency and minimize measurement bias. The investigator applying the percussive massage was aware of the assigned intervention protocols but blinded to the specific study hypotheses.

### 2.4. Assessment Methods

#### 2.4.1. Anthropometric and Health Screening

Demographic data and anthropometric measurements were collected using standardized procedures during the first laboratory visit. A demographic questionnaire was administered to obtain basic participant information, including age, training experience, dominant leg, and health status. The participants were screened for recent injuries, musculoskeletal discomfort, or medical conditions that could affect neuromuscular performance. Body height and body mass were measured using a calibrated digital stadiometer and scale (Seca Model 769, Hamburg, Germany), and the Body Mass Index (BMI) was subsequently calculated. Additionally, to ensure physiological readiness prior to testing, a subjective “Recovery Score” was recorded for each participant.

#### 2.4.2. Chronotype

Chronotype was assessed using the Morningness–Eveningness Questionnaire (MEQ), originally developed by Horne and Östberg (1976) and validated in Turkish by Pündük et al. (2005) [34,35]. The MEQ consists of 19 items (a combination of Likert-type and time-scale questions) designed to evaluate individual preferences for daily activities and sleep timing. Responses to most items are scored on a 4- or 5-point scale, whereas questions 1, 2, and 10 utilize a time-scale format with options divided into 15 min intervals across a defined daily schedule.

Total scores range from 16 to 86 and classify individuals into one of five chronotype categories: definitely morning type (70–86), moderately morning type (59–69), intermediate type (42–58), moderately evening type (31–41), and definitely evening type (16–30) [34,35]. The validity of the original questionnaire and its chronotype classification has been supported by diurnal variations in core body temperature [35].

#### 2.4.3. COD Performance

COD performance was assessed using the T-test and the Illinois Agility Test, both of which are widely used and validated measures of change-of-direction speed.

T-Test: A cone configuration was set up forming a “T” shape (10 m forward, 5 m to the left, and 5 m to the right). The participants sprinted forward, shuffled laterally to the left and right, and backpedaled to their starting positions.

Illinois Agility Test: Participants started from a prone position (chest on the floor), stood up, and sprinted through a slalom course marked by cones over a 10 m × 5 m area.

The timing was recorded to the nearest 0.01 s using electronic timing gates (Brower Timing Systems, Draper, UT, USA). The participants performed two trials for each agility test, and the best performance was used for statistical analysis.

Electronic timing gates are widely used in change-of-direction (COD) and sprint assessments and have been shown to provide highly reliable and precise timing measurements in athletic populations [36]. Moreover, the COD tests selected in the present study demonstrated excellent test–retest reliability in team-sport athletes. Specifically, the T-Test has been reported to show excellent reliability with intraclass correlation coefficients (ICCs) ranging from 0.95 to 0.98 and low typical error [37], while the Illinois change-of-direction/agility test has also demonstrated high reliability (ICC ≈ 0.96) in male athletes [38]. These established reliability characteristics support the interpretation that the protocol-related changes observed in the present study are unlikely to be attributable solely to measurement errors when standardized testing procedures are applied.

#### 2.4.4. Vertical Jump and Neuromuscular Performance

Vertical jump performance and lower-limb kinetic characteristics were assessed using the Countermovement Jump (CMJ) test via the My Jump Lab application (v.4.4.2, developed by Dr. Carlos Balsalobre). This smartphone-based application uses high-speed video analysis to calculate jump variables based on the fundamental principles of Samozino’s method and has been validated against gold-standard force platforms [39].

Participants performed the CMJ from a stationary upright position with hands placed on the iliac crests to minimize arm swing contribution and standardize lower-limb force application. The protocol involved a controlled eccentric phase to a self-selected depth, immediately followed by a maximal concentric vertical jump. During the flight phase, participants were instructed to maintain knee extension and to land bilaterally with controlled ankle dorsiflexion to ensure consistency across trials. Three maximal attempts were performed, separated by a standardized 60 s rest interval, and the highest jump height was retained for analysis. Kinetic and kinematic variables—including jump height (cm), net vertical impulse (N·s), peak force (N), relative force (N·kg^−1^), take-off velocity (m·s^−1^), mean velocity (m·s^−1^), peak power (W), relative power (W·kg^−1^), and flight time (ms)—were calculated using the application’s validated model-based algorithms derived from the Samozino method.

The validity and reliability of the measurement tools were established based on existing literature. The My Jump 2 application has demonstrated excellent concurrent validity and test–retest reliability (ICC = 0.997, *p* < 0.001) compared to gold-standard force platforms, as reported by Balsalobre-Fernández et al. [39]. Such high reliability ensures that observed performance fluctuations reflect genuine physiological adaptations rather than measurement error. However, it should be noted that kinetic and kinematic variables—specifically force, velocity, impulse, and power—derived from the My Jump Lab application are model-based estimates utilizing the validated Samozino force–velocity method, rather than direct ground reaction force measurements. While previous validation studies report excellent agreement with force-platform data and a minimal standard error of estimate—approximately 1–2 cm for jump height—these variables should nonetheless be interpreted as indirect kinetic estimates [39,40]. This distinction is critical as model-derived calculations, despite their high correlation with direct measurements, rely on mathematical assumptions inherent to the Samozino method.

### 2.5. Statistical Analyses

All statistical analyses were performed using Python 3.12 (with libraries scipy, numpy, pandas, and statsmodels) and IBM SPSS Statistics for Windows, version 26.0 (IBM Corp., Armonk, NY, USA). Prior to main analyses, the normality of data distributions was assessed using the Shapiro–Wilk test. As the normality assumption was violated for most variables (*p* < 0.05), non-parametric methods were applied throughout. Descriptive statistics are presented as mean ± standard deviation (SD) and median (interquartile range, IQR) for continuous variables, and as frequencies and percentages for categorical variables. To evaluate the main effects of time of day (morning vs. evening), protocol (NM, L1, L3), and their interaction on performance outcomes, a series of two-way permutational analyses of variance (PERMANOVAs) was conducted separately for each dependent variable (univariate permutational approach), using Euclidean distance as the dissimilarity measure with 9999 permutations. To account for the repeated-measures structure of the crossover design, permutations were constrained within subjects (subject-level blocking), thereby preserving the within-subject dependency structure across conditions and time points. This restricted permutation scheme ensures that the exchangeability assumption underlying PERMANOVA is maintained in within-subject designs, and is conceptually analogous to a permutation-based repeated-measures ANOVA. PERMANOVA was selected due to its robustness to non-normality and heteroscedasticity, which were present in the current dataset [41,42,43]. A univariate PERMANOVA was preferred over a standard repeated-measures ANOVA not only due to non-normal data distribution, but because restricting permutations within subjects provides a more robust control for the non-independence of repeated measures in a crossover design, thereby avoiding the strict sphericity assumptions required by parametric tests. This within-subject permutation constraint was strictly applied for all experimental outcomes. Effect sizes for PERMANOVA are reported as partial omega squared (ωp^2^), estimated from the permutational sum-of-squares partitioning using the formula: ωp^2^ = (SS_effect − df_effect × MS_error)/(SS_total + MS_error). Interpretation thresholds were applied as small (0.01), medium (0.06), and large (0.14) according to established guidelines [44]. For variables showing significant protocol main effects or Protocol × Time interactions (*p* < 0.05), post hoc pairwise comparisons were performed using the Wilcoxon signed-rank test (two-tailed) to compare protocols within each time point (NM vs. L1, NM vs. L3, L1 vs. L3) and time points within each protocol (morning vs. evening). To control for multiple comparisons, the Bonferroni correction was applied, setting the significance threshold at *p* < 0.0167 for three pairwise contrasts per variable [45]. For Wilcoxon signed-rank post hoc tests, effect sizes are reported both as rank-biserial correlation (r = Z/√N), a distribution-free effect size measure, and as Cohen’s d for comparability with the broader sports science literature. Cohen’s d was computed as the standardized mean difference divided by the pooled standard deviation. Effect sizes for Wilcoxon tests are reported as Cohen’s d, interpreted as small (0.2–0.5), medium (0.5–0.8), and large (>0.8) [46].

## 3. Results

Participant demographic and anthropometric characteristics are presented in Table 1. Eighteen trained male soccer players volunteered for the study (mean age 21.22 ± 1.93 years; height 178.72 ± 7.51 cm; body mass 69.06 ± 9.30 kg; BMI 21.56 ± 2.03 kg/m^2^). Mean training experience was 3.61 ± 1.20 years, indicating a moderately trained cohort. The mean Morningness–Eveningness Questionnaire (MEQ) score was 49.17 ± 6.54, classifying participants as intermediate chronotype.

Descriptive statistics for change-of-direction (COD) performance and countermovement jump kinetic variables are presented in Table 2. Two-way PERMANOVA analyses revealed significant main effects of protocol for T-test performance, vertical jump height, peak and relative force, peak and relative kinetic output, mean velocity, take-off velocity, net vertical impulse, and flight time (all *p* < 0.001), whereas no significant protocol effect was observed for the Illinois COD Test (*p* = 0.352). Significant main effects of time of day were identified for most variables (*p* ≤ 0.010), indicating diurnal variation in performance outcomes.

Effect sizes for protocol were large across several kinetic and velocity-related variables (ωp^2^ = 0.56–0.84), particularly for vertical jump height (ωp^2^ = 0.84), relative force (ωp^2^ = 0.73), and relative kinetic output (ωp^2^ = 0.72). Time-of-day effects demonstrated medium-to-large effect sizes, consistent with lower morning performance values under control conditions. Significant Protocol × Time interactions were observed for vertical jump height (*p* = 0.004), peak vertical force (*p* = 0.036), relative force (*p* = 0.032), and net vertical impulse (*p* = 0.021), with corresponding medium effect sizes (ωp^2^ = 0.16–0.26). These interactions indicate that the magnitude of protocol-related performance changes differed between morning and evening sessions.

Post hoc pairwise comparisons (Wilcoxon signed-rank test with Bonferroni adjustment) for variables demonstrating significant protocol main effects or interactions are presented in Table 3 and Table 4. Table 3 summarizes morning comparisons relative to the no-massage morning condition, while Table 4 summarizes evening comparisons relative to the no-massage evening condition. Effect sizes are reported as Cohen’s d (small 0.2–0.5, medium 0.5–0.8, large > 0.8).

In morning sessions (Table 3), the moderate-intensity condition (L3) was associated with significantly greater values than the no-massage condition across multiple kinetic variables, including relative force (d = 1.61), net vertical impulse (d = 1.69), and peak kinetic output (d = 1.33) (all *p* < 0.001). The low-intensity condition (L1) demonstrated smaller or non-significant differences in several force-related variables (e.g., peak force *p* = 0.142), although improvements in jump height (d = 1.63, *p* < 0.001) and velocity-related measures were observed. Direct comparisons between L1 and L3 indicated statistically significant differences in selected kinetic variables (e.g., peak force d = 0.57, *p* < 0.001), suggesting a greater magnitude of change under the moderate-intensity protocol.

In evening sessions (Table 4), both percussive massage intensities were associated with significantly greater values compared with the no-massage condition across several kinetic and jump-related variables (all *p* < 0.001), with large standardized effect sizes (e.g., L3 relative force d = 2.89; jump height d = 3.19). Comparisons between L3 and L1 demonstrated statistically significant differences in multiple kinetic parameters (d = 0.79–0.89, *p* < 0.001), indicating a greater magnitude of response under the moderate-intensity protocol. In contrast, Illinois COD performance did not show significant responsiveness (*p* > 0.05), suggesting limited sensitivity of this test to acute force–time-related changes. Overall, the findings indicate a diurnal variation in performance outcomes, with lower baseline values observed in morning sessions relative to evening sessions. Both percussive massage conditions were associated with improvements across time-of-day conditions; however, the moderate-intensity application (35 Hz) demonstrated comparatively larger effects, particularly during morning sessions.

## 4. Discussion

The primary aim of this study was to examine the acute effects of two percussive massage (PM) intensities (28 Hz and 35 Hz) on change-of-direction (COD) performance, vertical jump outcomes, and model-derived kinetic variables in trained male football players, while accounting for potential time-of-day influences. The findings indicate that both PM intensities were associated with improvements in COD performance and vertical jump-related variables compared with the no-massage condition. Notably, several force–time characteristics, including net vertical impulse and force-related metrics, demonstrated significant protocol × time-of-day interactions. These interaction effects suggest a time-dependent modulation of the PM response, with the moderate-intensity condition (35 Hz) showing comparatively larger performance changes during morning sessions.

Consistent with well-established circadian patterns, baseline performance in the no-massage condition was generally lower in the morning than in the evening across most explosive and kinetic variables. Diurnal variations in neuromuscular performance have been widely attributed to fluctuations in core body temperature, neural drive, and hormonal profiles, particularly elevated morning cortisol levels and reduced muscle temperature [19,20,21,22,23,24,25]. The present findings align with this literature, confirming a clear “morning slump” in COD speed and jump-related variables under control conditions. Critically, this study provides a novel contribution by demonstrating that acute PM application, specifically at 35 Hz, effectively mitigates the observed circadian-related performance decline in this homogeneous cohort.

The observation that moderate-intensity PM was particularly effective during morning sessions suggests that PM may act as a neuromuscular priming strategy capable of partially compensating for suboptimal physiological readiness early in the day. Mechanical vibration delivered via percussive devices has been shown to increase local blood flow, elevate muscle temperature, and enhance afferent feedback from muscle spindles, thereby facilitating neural activation [4,5,16]. These mechanisms may be especially beneficial in the morning, when baseline neuromuscular activation and muscle–tendon readiness are reduced. In this context, PM appears to function not merely as a general warm-up adjunct, but as a time-specific intervention targeting circadian vulnerability.

The improvements observed in vertical jump height, velocity-related variables, and force–time characteristics following PM are in agreement with recent systematic reviews reporting acute benefits of percussive therapy on neuromuscular performance without detrimental effects on strength or power output [4,7]. Konrad et al. [5] demonstrated that PM applied with a Hypervolt device increased range of motion without impairing muscle performance, suggesting favorable alterations in muscle viscoelastic properties. Similarly, Ferreira et al. [7] reported that the combined percussion and vibration stimulus can reduce passive muscle stiffness while preserving or enhancing functional performance. These thixotropic effects—characterized by reductions in muscle viscosity and passive resistance—may facilitate more efficient movement execution during explosive tasks by lowering internal tissue friction and improving contraction velocity.

Although reductions in muscle stiffness are sometimes considered potentially unfavorable for stretch–shortening cycle efficiency, it is essential to differentiate between passive viscoelastic stiffness and active force-transmission capacity. Existing evidence suggests that percussive massage primarily influences passive tissue properties rather than the active elastic components responsible for force storage and return [5,15,33]. In the present study, concurrent increases in jump height, take-off velocity, and net vertical impulse indicate that the intervention was not associated with reductions in effective concentric force application. While tissue compliance was not directly measured, these performance changes suggest that any alterations in passive mechanical properties did not impair, and may have been compatible with, efficient force–time characteristics during the concentric phase. This interpretation aligns with prior findings reporting improved movement velocity following percussive massage interventions [18], potentially reflecting altered neuromuscular activation patterns or reduced passive mechanical resistance.

A key finding of this study is the clear dose-dependent response observed for kinetic variables. While both PM intensities improved gross performance outcomes such as COD speed and jump height, only the moderate-intensity protocol (35 Hz) consistently produced large improvements in force- and impulse-related variables, particularly during morning sessions. This aligns with foundational work demonstrating that vibration frequencies in the range of 30–40 Hz optimally stimulate muscle spindle Ia afferents and the tonic vibration reflex, thereby enhancing motor unit recruitment and force production [16,47]. Systematic evidence further supports the notion that higher PM intensities are more effective for eliciting neuromuscular potentiation, whereas lower intensities may primarily facilitate tissue mobilization [4].

The COD findings warrant specific consideration. The T-Test demonstrated significant improvements following PM, whereas the Illinois COD Test did not show protocol-related changes. This discrepancy is likely attributable to differences in task demands. The Illinois test involves a longer duration and greater metabolic contribution, placing increased emphasis on anaerobic capacity and fatigue resistance rather than purely neuromuscular explosiveness [48,49,50].

The absence of significant findings in the Illinois COD test should also be interpreted with consideration of measurement sensitivity. Given its longer duration and greater metabolic contribution, the test may be less sensitive to detect small acute neuromuscular potentiation effects compared to acceleration-dominant tasks. Therefore, the lack of statistical significance may reflect sensitivity limitations rather than a complete absence of physiological influence.

As PM is primarily hypothesized to enhance short-duration, ATP–PC–dominated actions through neuromuscular priming and viscoelastic modulation, its effects may be less pronounced in longer, metabolically demanding COD tasks. This interpretation is consistent with prior research suggesting that PM is more effective for brief explosive actions than for sustained high-intensity efforts [4,15]. Future studies employing shorter COD assessments, such as the 505 test, may provide greater sensitivity to PM-induced neuromuscular changes [51].

It should be noted that all kinetic variables in the present study were derived using a validated model-based approach rather than direct force-platform measurements [39]. Although this method has demonstrated strong validity and reliability, the calculated outputs represent biomechanical estimates rather than direct ground reaction force data. Given the central role of force–time integration in acceleration and movement initiation, particular attention may be directed toward variables such as net vertical impulse and relative force, which are closely linked to changes in velocity according to fundamental mechanical principles. In this context, the present findings highlight the utility of impulse-related metrics when interpreting acute performance modifications, while acknowledging that model-derived power outputs provide complementary, rather than primary, information.

Several limitations should be acknowledged. The sample consisted exclusively of young male football players, limiting generalizability to female athletes or other populations. Only acute responses were examined; thus, the duration of PM-induced potentiation remains unknown. Additionally, underlying physiological mechanisms such as electromyographic activity or intramuscular temperature were not directly measured. The absence of a sham condition represents another limitation; although passive rest was used as a control, expectancy effects cannot be fully excluded due to the tactile nature of PM. Finally, all participants exhibited an intermediate chronotype, which likely reduced inter-individual circadian variability but limits extrapolation to athletes with extreme morning or evening preferences.

An additional limitation relates to potential expectancy and placebo effects. Due to the tactile and perceptible nature of percussive massage, full participant blinding was not feasible. Although participants were unaware of the specific hypotheses and relative intensity expectations, the absence of a sham condition (e.g., device contact without oscillatory stimulus) limits the ability to fully dissociate physiological neuromuscular mechanisms from expectancy-driven performance facilitation. It is possible that perceptual stimulation and anticipatory beliefs contributed, at least in part, to acute performance enhancements. Future sham-controlled studies are warranted to further isolate underlying mechanisms.

Despite these limitations, the present findings suggest that percussive massage may represent a time-sensitive warm-up adjunct. Low-intensity PM was associated with improvements in COD performance and jump height, whereas the moderate-intensity condition (~35 Hz) demonstrated comparatively larger changes in force–time-related variables, including impulse. These effects were particularly evident during morning sessions, when baseline performance values were lower relative to evening conditions. Although subject-level blocking was applied to account for within-subject dependency, the use of PERMANOVA in a repeated-measures crossover design remains less conventional compared to parametric approaches, and readers should interpret the statistical outcomes with this context in mind.

## 5. Conclusions

The present study examined the acute effects of two percussive massage intensities on change-of-direction (COD) performance, vertical jump outcomes, and force–time-related variables in trained male football players. The findings indicate that both low-intensity (28 Hz) and moderate-intensity (35 Hz) percussive massage were associated with improvements in COD speed and jump height relative to a no-massage condition.

A time-dependent response pattern was observed for selected kinetic variables. Although both intensities were linked to performance enhancements, the moderate-intensity condition (35 Hz) demonstrated comparatively larger changes in force- and impulse-related measures. These effects were particularly evident during morning sessions, when baseline performance values were lower than evening levels.

The absence of significant changes in the Illinois COD test suggests that percussive massage may exert greater influence on short-duration, force–time-dependent tasks than on longer-duration assessments involving greater metabolic contribution.

From a practical standpoint, short-duration percussive massage may be considered as a warm-up adjunct, particularly in contexts where early-morning sessions are performed. However, interpretation of effect magnitude should account for the homogeneous sample and acute crossover design. Future research should explore longer-term adaptations, include diverse chronotypes, and incorporate direct mechanical measurements to further clarify underlying mechanisms.

## Figures and Tables

**Table 1:** Participant Characteristics.

Variable	Mean ± SD	Range
Age (years)	21.22 ± 1.93	18–25
Height (cm)	178.72 ± 7.51	170–194
Body mass (kg)	69.06 ± 9.30	53–82
BMI (kg/m^2^)	21.56 ± 2.03	17.51–25.03
Training experience (years)	3.61 ± 1.20	2–6
MEQ score	49.17 ± 6.54	40–60

**Table 2:** Descriptive Statistics and PERMANOVA Results for Change-of-Direction (COD) and Countermovement Jump Variables.

Variable	NM Morning	NM Evening	L1 Morning	L1 Evening	L3 Morning	L3 Evening	Time	Protocol	Interaction	ES (ω_p^2^)
T-Test (COD) (s) †	10.92 (1.02)	10.78 (1.15)	10.40 (0.82)	10.25 (0.68)	10.58 (0.95)	10.50 (0.88)	0.005	<0.001	0.125	0.43 (large)
Illinois COD Test (s) †	16.20 (1.45)	16.10 (1.30)	15.95 (0.75)	15.80 (1.10)	16.15 (0.90)	16.00 (0.80)	0.215	0.352	0.478	0.06 (small)
Jump Height (cm)	33.50 (6.80)	34.20 (6.10)	36.00 (6.20)	37.50 (5.00)	37.50 (4.50)	39.00 (3.80)	<0.001	<0.001	0.004	0.84 (large)
Peak Vertical Force (N)	1180 (75)	1190 (72)	1240 (80)	1260 (65)	1290 (55)	1320 (45)	0.003	<0.001	0.036	0.33 (medium)
Relative Force (N/kg)	14.00 (0.90)	14.10 (0.85)	14.60 (0.95)	14.90 (0.70)	15.10 (0.60)	15.50 (0.50)	<0.001	<0.001	0.032	0.73 (large)
Peak Kinetic Output (W)	1550 (240)	1580 (220)	1730 (250)	1820 (200)	1880 (160)	1980 (130)	<0.001	<0.001	0.112	0.64 (large)
Relative Kinetic Output (W/kg)	18.30 (3.00)	18.70 (2.80)	20.30 (3.10)	21.20 (2.50)	22.00 (2.00)	23.20 (1.70)	0.004	<0.001	0.098	0.72 (large)
Mean Velocity (m/s)	1.30 (0.15)	1.32 (0.14)	1.38 (0.16)	1.44 (0.12)	1.45 (0.10)	1.50 (0.08)	0.010	<0.001	0.145	0.67 (large)
Take-off Velocity (m/s)	2.60 (0.28)	2.65 (0.25)	2.75 (0.28)	2.85 (0.22)	2.90 (0.18)	3.00 (0.15)	0.010	<0.001	0.132	0.66 (large)
Net Vertical Impulse (N·s)	220 (25)	225 (22)	235 (24)	242 (18)	248 (15)	255 (12)	<0.001	<0.001	0.021	0.56 (large)
Flight Time (ms)	530 (55)	540 (50)	565 (55)	580 (45)	595 (35)	610 (30)	0.010	<0.001	0.128	0.67 (large)

Values are median (IQR); † lower values indicate better performance. *p* < 0.05. ES = partial omega squared (ω_p^2^). Kinetic Output refers to derived power.).

**Table 3:** Post hoc Comparisons—Morning Changes (No-Massage Morning Baseline).

Variable	L1 vs. NM		L3 vs. NM		L1 vs. L3	
	*p*	d	*p*	d	*p*	d
T-Test (COD) (s) †	<0.001	0.67	0.007	0.54	0.832	-
Jump Height (cm)	<0.001	1.63	<0.001	1.72	0.003	0.63
Peak Vertical Force (N)	0.142	-	<0.001	0.96	<0.001	0.57
Relative Force (N/kg)	0.004	0.38	<0.001	1.61	<0.001	0.5
Peak Kinetic Output (W)	0.119	0.16	<0.001	1.33	<0.001	0.63
Relative Kinetic Output (W/kg)	0.002	0.46	<0.001	1.71	0.036	0.34
Mean Velocity (m/s)	0.002	0.4	<0.001	1.75	0.038	0.29
Take-off Velocity (m/s)	0.002	0.39	<0.001	1.71	0.027	0.31
Net Vertical Impulse (N·s)	0.130	-	<0.001	1.69	0.001	0.58
Flight Time (ms)	0.002	0.4	<0.001	1.71	0.027	0.3

† lower values indicate better performance.

**Table 4:** Post hoc Comparisons—Evening Changes (No-Massage Evening Baseline).

Variable	L1 vs. NM		L3 vs. NM		L1 vs. L3	
	*p*	d	*p*	d	*p*	d
T-Test (COD) (s) †	<0.001	1.13	0.007	1.05	0.832	-
Jump Height (cm)	<0.001	1.71	<0.001	3.19	0.003	0.80
Peak Vertical Force (N)	0.142	-	<0.001	3.15	<0.001	0.85
Relative Force (N/kg)	<0.001	1.57	<0.001	2.89	<0.001	0.89
Peak Kinetic Output (W)	0.119	-	<0.001	3.16	<0.001	0.85
Relative Kinetic Output (W/kg)	<0.001	1.72	<0.001	3.15	0.036	0.85
Mean Velocity (m/s)	<0.001	1.70	<0.001	3.03	0.038	0.79
Take-off Velocity (m/s)	<0.001	1.75	<0.001	3.03	0.027	0.82
Net Vertical Impulse (N·s)	0.130	-	<0.001	3.08	0.001	0.81
Flight Time (ms)	<0.001	1.74	<0.001	3.08	0.027	0.81
Illinois COD Test (s) †	0.569	-	0.551	-	0.005	0.16

† lower values indicate better performance.

## Data Availability

Data are available for research purposes from the corresponding author upon reasonable requests.

## Abbreviations

The following abbreviations are used in this manuscript:

CMJ	countermovement jump
PM	percussive massage
TVR	tonic vibration reflex
